# Association of circulating adiponectin and leptin levels with the risk of diabetic peripheral neuropathy

**DOI:** 10.3389/fendo.2024.1505082

**Published:** 2024-12-13

**Authors:** Zongcun Chen, Shasha Fu, Shuchang Lai, Maoxiong Fu, Guankui Du

**Affiliations:** ^1^ Department of Endocrinology, The Second Affiliated Hospital of Hainan Medical University, Haikou, China; ^2^ Key Laboratory of Tropical Translational Medicine of Ministry of Education, School of Basic Medicine and Life Sciences, Hainan Medical University, Haikou, China; ^3^ Department of Biochemistry and Molecular Biology, Hainan Medical University, Haikou, China; ^4^ Department of Respiratory and Critical Care Medicine, Haikou Affiliated Hospital of Central South University Xiangya School of Medicine (Haikou People’s Hospital), Haikou, China

**Keywords:** diabetic peripheral neuropathy, adiponectin, leptin, association, case-control study

## Abstract

**Background:**

Adipokines have been implicated in the pathogenesis of type 2 diabetes mellitus (T2DM) and related complications due to their roles in metabolic regulation and inflammation. However, the relationship between these adipokines and diabetic peripheral neuropathy (DPN) remains unclear.

**Methods:**

A case-control study was performed with 198 patients with DPN and 205 T2DM patients without DPN from the Endocrinology Department at the Second Affiliated Hospital of Hainan Medical University. Circulating adiponectin and leptin levels were quantified via enzyme-linked immunosorbent assays. Logistic regression models, adjusting for age, sex, BMI, smoking status, and diabetes duration, were applied to evaluate the associations between adiponectin and leptin levels and DPN risk.

**Results:**

DPN patients exhibited lower adiponectin (P=0.001) and higher leptin (P=0.007) levels than diabetic controls. Confounders-adjusted analyses revealed that higher adiponectin levels correlated with reduced DPN risk (OR, tertile 3 vs. tertile 1: 0.52; 95% CI: 0.30-0.90), whereas elevated leptin levels were linked to increased DPN risk (OR, tertile 3 vs. tertile 1: 1.91; 95% CI: 1.10-3.32). Stratified analyses confirmed consistent findings across subgroups without statistically significant interactions.

**Conclusions:**

Circulating adiponectin and leptin levels correlate with DPN risk in diabetic patients, suggesting their potential as biomarkers for high-risk DPN identification and guiding targeted prevention and management.

## Introduction

Diabetic peripheral neuropathy (DPN), a prevalent diabetic complication affecting half of all diabetic patients, is marked by peripheral nerve damage, primarily in the limbs (1). This condition significantly impacts patient well-being through chronic pain, sensory deficits, and heightened risks of foot complications, imposing substantial healthcare costs and diminished quality of life (2). Given the incomplete understanding of its pathophysiology and the scarcity of efficacious management strategies, the identification of modifiable DPN risk factors is imperative for tailoring preventive and therapeutic interventions aimed at curbing its incidence and severity (3, 4).

The potential role of adipokines, including adiponectin and leptin, in the development of diabetic complications like DPN is gaining recognition due to their involvement in metabolic regulation and inflammatory processes, key factors in diabetes and its sequelae (5). Adiponectin, known for its anti-inflammatory and insulin-sensitizing effects, offers protection against conditions such as atherosclerosis and type 2 diabetes (6, 7). Conversely, leptin, with its pro-inflammatory characteristics, often elevated in obesity and type 2 diabetes, contributes to insulin resistance and metabolic dysfunction (6, 7). Given these roles, adiponectin and leptin may influence DPN development.

Several studies have explored the link between adiponectin and DPN risk among patients with type 2 diabetes, yielding mixed results: some report an inverse association (8, 9), others find no significant correlation (10) or even a positive link (11, 12). Research on leptin and DPN is limited and also inconsistent (11, 13, 14). Prior investigations often suffer from small sample sizes (8, 10, 11, 13, 14), lack of adjustment for confounders (8, 10, 13, 14), or aggregation of various diabetic complications into a single outcome variable (12). Therefore, relationship between adiponectin and leptin levels and DPN risk requires further investigations.

This study aims to investigate the relationship between circulating levels of adiponectin and leptin and the risk of developing DPN in individuals with diabetes. By elucidating these associations, our study might contribute to the growing body of evidence on the role of adipokines in diabetic complications and inform the development of strategies for the prevention and management of DPN.

## Methods

### Study population

This case-control study was conducted on type 2 diabetes mellitus (T2DM) patients, recruited from the Endocrinology Department at the Second Affiliated Hospital of Hainan Medical University, China, between August 2019 and December 2022. A total of 198 patients diagnosed with diabetic peripheral neuropathy (DPN group) and 205 diabetic patients without neuropathy (diabetic control group) were recruited. The DPN group and the diabetic control group were frequency matched by 5-year age groups and sex. All participants were diagnosed with T2DM according to the American Diabetes Association criteria (15). Participants were excluded if they had significant comorbidities such as major cardiovascular disease, infectious and inflammatory conditions, severe renal or hepatic impairment or cancer. The study protocol was reviewed and approved by the hospital’s ethics committee, and written informed consent was obtained from all participants.

### DPN diagnosis

Diagnosis of DPN was determined through a comprehensive approach encompassing clinical symptom evaluation and neurological assessments. Key assessments included the identification of neuropathic symptoms (numbness, tingling, pain), examination of ankle reflexes, and sensory testing using a 128-Hz tuning fork for vibration perception and a 10-g monofilament for light touch sensitivity. A diagnosis of DPN was established if patients without clinical symptoms presented with at least two abnormalities in neurological assessments, or if those with symptoms showed at least one abnormality (16). This diagnostic protocol ensured a thorough and accurate assessment of DPN status among participants.

### Data collection

Demographic data, including age, sex, smoking status were obtained via a self-reported questionnaire. Clinical data on height, weight, hypertension and duration of diabetes were extracted from medical records. Body mass index (BMI) was calculated as the weight (kg) divided by the height squared (m^2^). Comprehensive laboratory assessments conducted at the time of recruitment provided vital biochemical data, including levels of glycated hemoglobin (HbA1c), total cholesterol (TC), triglycerides (TG), high-density lipoprotein cholesterol (HDL-C), and low-density lipoprotein cholesterol (LDL-C). These laboratory results were accessed through the hospital’s sophisticated laboratory information system, ensuring accuracy and reliability of the biochemical data.

### Measurements of adiponectin and leptin

Fasting blood samples were collected from all participants using EDTA tubes and processed within two hours of collection. Plasma samples were separated by centrifugation at 3000 rpm for 15 minutes and stored at -80°C until analysis. The circulating levels of adiponectin and leptin were measured using commercial enzyme-linked immunosorbent assay (ELISA) kits (CUSABIO, China) following the manufacturer’s instructions. The minimum detectable levels of adiponectin and leptin were both 0.156 ng/ml. The intra-assay and inter-assay coefficients of variation for the two assays were below 8% and 10%, respectively.

### Statistical analysis

Descriptive statistics were used to summarize the characteristics of the study population, with continuous variables presented as means ± standard deviations or medians with interquartile ranges, and categorical variables expressed as frequencies and percentages. Comparisons between the DPN and diabetic control groups were performed using the chi-square test for categorical variables and the independent t-test or Mann-Whitney U test for continuous variables, depending on the data distribution.

To examine the association between circulating adiponectin and leptin levels and the risk of DPN, logistic regression models were employed. Odds ratios (ORs) with 95% confidence intervals (CIs) were calculated, adjusting for potential confounders including age, sex, educational level, BMI, smoking status and duration of diabetes. The levels of adiponectin and leptin were analyzed both as continuous variables and as categorical variables (divided into tertiles based on the distribution in the study population). Stratified analysis for associations of adiponectin and leptin levels with DPN were performed by patients’ characteristics, and interaction analysis were also conducted by including the cross-product term in the logistic regression models.

Statistical significance was defined as a two-tailed p-value of less than 0.05. All analyses were performed using SPSS software, version 22.0 (SPSS Inc., Chicago, IL, USA).

## Results

### DPN and control groups matched for demographic characteristics

The characteristics of the participants are presented in **Table 1**. The distributions of age, sex, educational levels, body mass index (BMI), and smoking status were comparable between the DPN group and diabetic control group (P > 0.05 for all comparisons). Glycemic control, as indicated by HbA1c levels, was significantly poorer in the DPN group (8.7 ± 2.0%) compared to the control group (8.3 ± 1.8%, P = 0.030). The levels of TG, but not other lipid indications, were higher in DPN group (1.89 ± 1.18 mmol/L) than those in the control group (1.67 ± 1.05 mmol/L, P=0.049). Additionally, the DPN group had a significantly longer duration of diabetes (median: 11.7 years [IQR: 8.5, 16.8]) compared to the control group (median: 7.3 years [IQR: 5.0, 11.8], P < 0.001).

**Table 1:** Patients’ characteristics.

Variables	DPN patients N=198	Diabetic controls N=205	P
Age (years) ^a^	62.4 ± 8.9	62.7 ± 9.1	0.705
Sex ^b^			0.861
Male	91 (46.0)	96 (46.8)	
Female	107 (54.0)	109 (53.2)	
Education level ^b^			0.366
Primary school or below	68 (34.3)	62 (30.2)	
Junior middle school	76 (38.4)	74 (36.1)	
Senior middle school or above	54 (27.3)	69 (33.7)	
BMI (kg/m^2^) ^a^	23.4 ± 3.2	23.6 ± 2.9	0.484
Smoking ^b^			0.573
No	129 (65.2)	139 (67.8)	
Yes	69 (34.8)	66 (32.2)	
HbA1c (%) ^a^	8.7 ± 2.0	8.3 ± 1.8	0.030
TC (mmol/L) ^a^	5.27 ± 1.24	5.06 ± 1.12	0.075
TG (mmol/L) ^a^	1.89 ± 1.18	1.67 ± 1.05	0.049
LDL-C (mmol/L) ^a^	2.94 ± 0.95	2.92 ± 0.91	0.830
HDL-C (mmol/L) ^a^	1.16 ± 0.41	1.17 ± 0.38	0.809
Hypertension ^b^			0.096
No	85 (42.9)	105 (51.2)	
Yes	113 (57.1)	100 (48.8)	
Diabetes duration (years) ^c^	11.7 (8.5, 16.8)	7.3 (5.0, 11.8)	<0.001
Adiponectin, ug/mL ^c^	8.5 (6.2, 11.3)	9.9 (7.0, 13.0)	0.001
Leptin, ng/ml ^c^	19.7 (12.3, 25.8)	15.8 (11.3, 23.5)	0.007

DPN, diabetic peripheral neuropathy; BMI, body mass index; HbA1c, glycated hemoglobin; TC, total cholesterol; TG, triglyceride; LDL-C, low-density lipoprotein cholesterol; HDL-C, high-density lipoprotein cholesterol.

^a^Data are presented as mean ± SD, and comparisons between the groups were performed using a t test. ^b^Data are presented as n (%), and compared using χ2 test. ^c^Data are presented as median (25^th^ percentile, and 75^th^ percentile), and compared using a Mann-Whitney U test.

### DPN group had lower adiponectin and higher leptin levels

**Figure 1:**
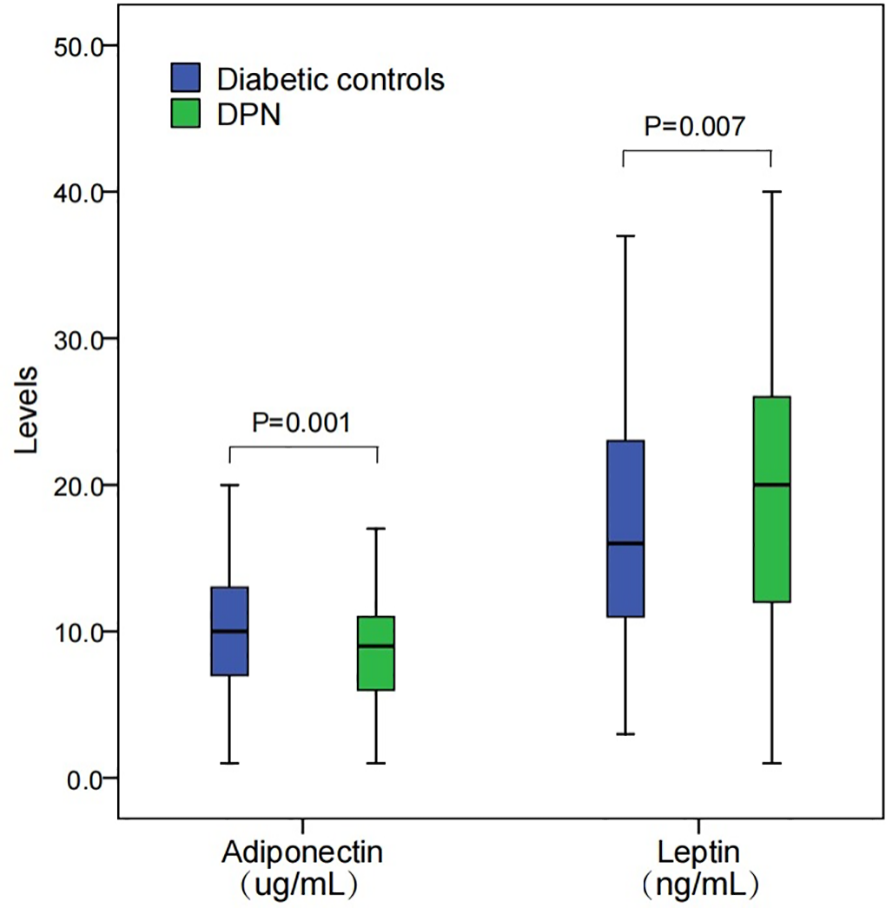
Levels of adiponectin and leptin among DPN patients and diabetic controls. DPN, diabetic peripheral neuropathy. Levels between groups were compared using a Mann-Whitney U test.

The box plot presented in **Figure 1** illustrates the distribution of adiponectin and leptin levels among two groups. For adiponectin, the levels in DPN patients (median, 8.5 ug/mL; interquartile, 6.2-11.3 ug/mL) were significantly lower compared to those in diabetic controls (median, 9.9 ug/mL; interquartile, 7.0, 13.0 ug/mL; P=0.001). Regarding leptin, the levels are shown to be higher in DPN patients (median, 19.7 ng/mL; interquartile, 12.3-25.8 ng/mL) than in diabetic controls (median, 15.8 ng/mL; interquartile, 11.3-23.5 ng/mL; P=0.007).

### Continuous increase in adiponectin levels decreased DPN risk

The association between circulating adiponectin levels and the risk of DPN is shown in **Table 2**. In the unadjusted model, participants in the highest tertile of adiponectin had a significantly reduced risk of DPN compared to those in the lowest tertile (crude OR, 0.49; 95% CI, 0.30-0.80). After adjusting for potential confounders, including age, sex, education level, BMI, smoking status, and duration of diabetes, this association remained significant (adjusted OR, 0.52; 95% CI, 0.30-0.90). A trend analysis across the three tertiles also supports this association (P for trend = 0.020). Furthermore, a continuous increase in adiponectin levels was associated with a decreased risk of DPN (adjusted OR, 0.91 per unit increase; 95% CI: 0.86, 0.97).

**Table 2:** Association of adiponectin and leptin levels with DPN.

Variables	DPN n (%)	Diabetic controls n (%)	Unadjusted OR (95% CI)	Adjusted OR (95% CI)^a^
Adiponectin				
Tertile 1	77 (38.9)	58 (28.3)	1.00 (reference)	1.00 (reference)
Tertile 2	67 (33.8)	67 (32.7)	0.78 (0.48, 1.23)	0.67 (0.40, 1.13)
Tertile 3	54 (27.3)	80 (39.0)	0.49 (0.30, 0.80)	0.52 (0.30, 0.90)
P for trend			0.004	0.020
Continuous (per unit increase)			0.91 (0.86, 0.96)	0.91 (0.86, 0.97)
Leptin				
Tertile 1	55 (27.8)	80 (39.0)	1.00 (reference)	1.00 (reference)
Tertile 2	65 (32.8)	69 (33.7)	1.01 (0.63, 1.64)	1.13 (0.67, 1.94)
Tertile 3	78 (39.4)	56 (27.3)	1.74 (1.07, 2.82)	1.91 (1.10, 3.32)
P for trend			0.025	0.020
Continuous (per unit increase)			1.04 (1.01, 1.07)	1.05 (1.02, 1.08)

DPN, diabetic peripheral neuropathy; OR, odds ratio; CI, confidence interval.

^a^Adjusted for age, sex, education level, body mass index, smoking, and duration of diabetes.

### Higher leptin levels associated with increased DPN risk

Association with DPN risk for leptin levels appears to be opposite to that for adiponectin (**Table 2**). The unadjusted analysis indicated that participants in the highest tertile of leptin had significantly higher risk of DPN compared to those in the lowest tertile (crude OR, 1.74; 95% CI, 1.07-2.82). This association persisted after adjustment for confounders (adjusted OR, 1.91; 95% CI, 1.10-3.32). Additionally, each unit increase in leptin was associated with a 5% increase in the risk of DPN (adjusted OR, 1.05 per unit increase; 95% CI, 1.02-1.08).

### Subgroup-specific associations of adiponectin and leptin with DPN risk

**Table 3** presents the results of the stratified analysis by age, sex, education level, BMI, and smoking status to assess whether the association between adiponectin and leptin levels and DPN risk varied across these subgroups. For adiponectin, a significant reduction in DPN risk was found among participants with a primary school education or below who were in the highest adiponectin tertile (OR = 0.22, 95% CI: 0.07, 0.68), while the strength of association among participants with a junior middle school or above appeared weaker. However, the interaction between education level and adiponectin was not statistically significant (P for interaction = 0.649). Similarly, higher adiponectin levels were associated with a lower risk of DPN (Tertile 3: OR = 0.38, 95% CI: 0.19, 0.75) among non-smokers, but no significant association was found in smokers. The interaction between smoking status and adiponectin was not statistically significant (P for interaction = 0.363). For leptin, the association with DPN risk tends to be stronger among participants who were aged 60 years or younger, female or smoke than those in their corresponding comparison subgroups. However, the interaction terms were not statistically significant (P for interaction > 0.05). Overall, no significant interaction was observed between patients’ characteristics and adiponectin and leptin levels on DPN risk.

**Table 3:** Stratified analysis for associations between adipokines and DPN by patients’ characteristics.

Characteristics	Odds ratio (95% confidence interval) ^a^					
	Adiponectin			Leptin		
	Tertile 1	Tertile 2	Tertile 3	Tertile 1	Tertile 2	Tertile 3
Age						
≤60 years	Reference	0.90 (0.44, 1.88)	0.38 (0.16, 0.92)	Reference	1.78 (0.78, 4.08)	2.56 (1.06, 6.09)
>60 years	Reference	0.41 (0.18, 0.93)	0.45 (0.21, 0.93)	Reference	0.79 (0.38, 1.62)	1.49 (0.72, 3.10)
P for interaction	0.217			0.238		
Sex						
Female	Reference	0.45 (0.20, 1.01)	0.39 (0.17, 0.89)	Reference	0.97 (0.42, 2.20)	2.46 (1.09, 5.57)
Male	Reference	0.77 (0.37, 1.58)	0.55 (0.24, 1.22)	Reference	1.48 (0.72, 3.05)	1.40 (0.64, 3.06)
P for interaction	0.441			0.211		
Education level						
Primary school or below	Reference	0.34 (0.12, 0.96)	0.22 (0.07, 0.68)	Reference	1.33 (0.50, 3.53)	2.07 (0.78, 5.50)
Junior middle school or above	Reference	0.76 (0.41, 1.42)	0.64 (0.33, 1.23)	Reference	1.08 (0.57, 2.04)	1.82 (0.93, 3.55)
P for interaction	0.649			0.953		
BMI						
<24 kg/m^2^	Reference	0.51 (0.26, 1.01)	0.48 (0.23, 1.01)	Reference	1.04 (0.54, 1.97)	2.07 (1.01, 1.97)
≥24 kg/m^2^	Reference	0.91 (0.39, 2.13)	0.53 (0.22, 1.30)	Reference	1.29 (0.49, 3.40)	1.88 (0.74, 4.78)
P for interaction	0.470			0.801		
Smoking						
No	Reference	0.62 (0.33, 1.17)	0.38 (0.19, 0.75)	Reference	1.07 (0.56, 2.07)	1.69 (0.88, 3.24)
Yes	Reference	0.80 (0.30, 2.15)	1.29 (0.44, 3.86)	Reference	1.55 (0.59, 4.05)	2.86 (0.93, 8.81)
P for interaction	0.363			0.879		

BMI, body mass index.

^a^Adjusted for age, sex, education level, body mass index, smoking, and duration of diabetes.

## Discussion

### Summary of findings

In this study, we investigated the association between circulating levels of adiponectin and leptin with the risk of DPN. Our findings demonstrate that higher levels of adiponectin are associated with a lower risk of DPN, while higher levels of leptin are associated with an increased risk of DPN. These associations remained significant even after adjusting for potential confounders, including age, sex, body mass index (BMI), smoking, and duration of diabetes. These results highlight the potential role of adipokines in the pathogenesis of DPN and suggest that adiponectin and leptin may serve as valuable biomarkers for predicting the risk of DPN in diabetic patients.

### Comparison with previous studies

Previous studies have reported associations between adiponectin and diabetic complications among type 2 diabetes patients, although the evidence has been limited and inconsistent. In line with our finding, a study in China (mean age, ~55 years; mean BMI, ~24 kg/m^2^) found that plasma adiponectin levels were lower in DPN patients (n=90) that those non-DPN diabetic patients (n=90) (8). Similarly, a German study (mean age, ~72 years; mean BMI, ~31 kg/m^2^) of 43 DPN and 168 diabetic controls found that serum adiponectin levels were lower in the case group (9). However, an Indian study (mean age, ~54 years; mean BMI, ~26 kg/m^2^) of 43 DPN and 43 diabetic controls reported no association between serum adiponectin levels and DPN (10). Another study (mean age, ~60 years; mean BMI, ~26 kg/m^2^) of Chinese DPN (n=98) and non-DPN patients (n=121) reported that serum adiponectin levels were positively associated with DPN risk after adjusting for potential confounders (11). A study in India (mean age, ~50 years; mean BMI, ~25 kg/m^2^) also observed that serum adiponectin levels were higher in 138 diabetic neuropathy than those in 349 diabetic controls (12).

The positive association between leptin and DPN observed in our study is also consistent to a previous Chinese study (mean age, ~52 years; mean BMI, ~26 kg/m^2^) of 82 DPN and 200 diabetic controls, where higher levels of serum leptin were observed in the case group (13). In contrast, a Turkish study (mean age, ~57 years; mean BMI, ~28 kg/m^2^) found that the plasma leptin levels in diabetic patients with Sensorial neuropathy (n=38) or autonomic neuropathy (n=13) were not statistically different from those without these complications (14). In a Chinese study (mean age, ~60 years; mean BMI, ~26 kg/m^2^), serum leptin levels were positively associated with DPN risk when adjusting for age, sex, BMI, hypertension, HbA1c level, alcohol intake, smoking status, physical activity, and LDL, but this association was attenuated and became statistically insignificant when the regression model was further adjusted for lipid-lowering medications, eGFR, and disease duration (11).

The reason for the discrepancies in results across studies is unclear. It might be related to differences in population characteristics (e.g. age and BMI), genetic background and study design. For example, most of previous studies had small sample size (<100 cases) and did not consider confounders in the analysis. Further studies with better design are still needed to verify the relationship between adiponectin and leptin levels and DPN risk.

### Potential mechanisms underlying the associations

The observed associations between adiponectin, leptin, and the risk of DPN can be explained by several biological mechanisms. Adiponectin is known for its anti-inflammatory and insulin-sensitizing properties (6, 7), which may protect against the development of DPN by reducing systemic inflammation and improving glucose metabolism. Inflammation plays a central role in the pathogenesis of DPN, contributing to nerve damage and dysfunction (3). By attenuating inflammatory pathways, adiponectin may help preserve nerve function and prevent the onset of neuropathy. Additionally, adiponectin has been shown to enhance endothelial function and promote vascular health (17, 18), which could further contribute to its protective effects against DPN by ensuring adequate blood supply to peripheral nerves. Lower levels of adiponectin have been linked to increased oxidative stress (19), which is also contributor to nerve damage in diabetes. Leptin’s role in DPN also appears to be complex. Leptin has also been shown to promote the production of pro-inflammatory cytokines, such as TNF-α and IL-6, which contribute to nerve damage through inflammatory pathways (6, 7). Furthermore, leptin has been shown to impair endothelial function and promote atherogenesis (20, 21), potentially leading to microvascular complications that affect nerve health.

### Implications of findings

Our findings might have important implications for the understanding development of DPN. The adiponectin and leptin could be used as biomarkers for DPN risk. Measuring circulating levels of these adipokines could help identify individuals at higher risk for DPN, allowing for earlier intervention and more personalized management of diabetic patients. In addition, the inverse relationship between adiponectin levels and DPN risk suggests that increasing adiponectin levels could be a promising strategy for preventing or mitigating DPN in diabetic patients. Interventions that enhance adiponectin secretion or mimic its effects, such as lifestyle modifications (e.g., weight loss, physical activity) (22) or pharmacological agents (e.g., thiazolidinediones (23)), may hold potential in reducing the burden of DPN. Similarly, the positive association between leptin levels and DPN risk highlights the need for strategies to mitigate leptin resistance and reduce leptin levels in diabetic patients. This could be achieved through weight management, dietary interventions, or targeted therapies aimed at modulating leptin levels (24, 25). Further research is needed to validate these findings and explore the clinical utility of adiponectin and leptin measurements in routine practice.

### Limitations and strengths

While this study provides valuable insights into the associations between adiponectin, leptin, and DPN risk, it is important to acknowledge several limitations. First, the cross-sectional design of the study limits the ability to infer causality. Although the associations observed are biologically plausible and supported by previous research, longitudinal studies are needed to establish a temporal relationship between adipokine levels and the development of DPN. Second, the study population consisted of diabetic patients from a specific geographic region, which may limit the generalizability of the findings to other populations. Differences in genetic background, lifestyle factors, and healthcare practices could influence the relationship between adipokines and DPN.

Despite these limitations, the study has several strengths that enhance the validity of the findings. The sample size of our study is largest compared to previous studies. The use of a well-characterized cohort of diabetic patients with detailed clinical and biochemical assessments allowed for a comprehensive analysis of the associations between adipokines and DPN. The adjustment for multiple confounders, including age, sex, BMI, and duration of diabetes, helps to mitigate the potential for confounding.

## Conclusions

Our study provides evidence that higher circulating levels of adiponectin are associated with a reduced risk of DPN, while higher levels of leptin are associated with an increased risk. These findings suggest that adipokines play a significant role in the pathogenesis of DPN and may serve as useful biomarkers for identifying individuals at risk for this debilitating complication of diabetes. Future research should focus on longitudinal studies to confirm these associations and explore the underlying mechanisms.

## Data Availability

The original contributions presented in the study are included in the article/supplementary material. Further inquiries can be directed to the corresponding authors.
